# Use of mechanical airway clearance devices in the home by people with neuromuscular disorders: effects on health service use and lifestyle benefits

**DOI:** 10.1186/s13023-015-0267-0

**Published:** 2015-05-06

**Authors:** Trinity Mahede, Geoff Davis, April Rutkay, Sarah Baxendale, Wenxing Sun, Hugh JS Dawkins, Caron Molster, Caroline E Graham

**Affiliations:** Office of Population Health Genomics, Department of Health Western Australia, Perth, Australia; Data Linkage Branch, Department of Health Western Australia, Perth, Australia; Centre for Comparative Genomics, Murdoch University, Perth, Australia; Centre for Population Health Research, Curtin University of Technology, Perth, Australia; School of Pathology and Laboratory Medicine, University of Western Australia, Perth, Australia

**Keywords:** Neuromuscular disorder, Cough, Respiratory clearance, Mechanical in-exsufflation, Muscular dystrophy

## Abstract

**Background:**

People with neuromuscular disorders (NMD) exhibit weak coughs and are susceptible to recurrent chest infections and acute respiratory complications, the most frequent reasons for their unplanned hospital admissions. Mechanical insufflation-exsufflation (MI-E) devices are a non-invasive method of increasing peak cough flow, improving cough efficacy, the clearance of secretion and overcoming atelectasis. There is limited published evidence on the impact of home use MI-E devices on health service utilisation. The aims of the study were: to assess the self-reported health and lifestyle benefits experienced as a result of home use of MI-E devices; and evaluate the effects of in-home use of MI-E devices on Emergency Department (ED) presentations, hospital admissions and inpatient length of stay (LOS).

**Methods:**

Individuals with NMD who were accessing a home MI-E device provided through Muscular Dystrophy Western Australia were invited to participate in a quantitative survey to obtain information on their experiences and self-assessed changes in respiratory health. An ad-hoc record linkage was performed to extract hospital, ED and mortality data from the Western Australian Department of Health (DOHWA). The main outcome measures were ED presentations, hospital separations and LOS, before and after commencement of home use of an MI-E device.

**Results:**

Thirty seven individuals with NMD using a MI-E device at home consented to participate in this study. The majority (73%) of participants reported using the MI-E device daily or weekly at home without medical assistance and 32% had used the machine to resolve a choking episode. The survey highlighted benefits to respiratory function maintenance and the ability to manage increased health care needs at home. Not using a home MI-E device was associated with an increased risk of ED presentations (RR = 1.76, 95% CI 1.1-2.84). The number of hospital separations and LOS reduced after the use of MI-E device, but not significantly. No deaths were observed in participants using the MI-E device at home.

**Conclusions:**

Home use of a MI-E device by people living with NMD may have a potential impact on reducing their health service utilisation and risk of death. Future research with greater subject numbers and longer follow-up periods is recommended to enhance this field of study.

**Electronic supplementary material:**

The online version of this article (doi:10.1186/s13023-015-0267-0) contains supplementary material, which is available to authorized users.

## Background

Neuromuscular disorders (NMD) are a heterogeneous group of inherited or acquired diseases characterized by progressive muscle weakness and wasting which may affect either one or all of the major respiratory muscle groups (inspiratory, expiratory, and bulbar). Thus, respiratory deterioration is often the primary cause of both morbidity and mortality in people with NMD. As muscle weakness and wasting progress NMD patients lose the ability to cough effectively and clear respiratory secretions. This can lead to aspiration of salivary and oral contents, atelectasis, frequent recurrent respiratory infections, pneumonias and airway obstruction [1-4].

Respiratory complications in NMD had once been considered inevitable [5]. Advances in the management and treatment over the last 30 years have dramatically improved the overall morbidity and mortality in NMD [6,7]. Traditionally, the management of airway clearance involved intensive physiotherapy in combination with a ventilator and manually assisted coughing [8,9]. However, repeated intensive physiotherapy treatment is both labor-intensive and tiring for the patient. Mechanical insufflation-exsufflation (MI-E) devices, such as the CoughAssist™ device (Respironics INC, Murraysville, PA, USA) are a non-invasive alternative which have gained widespread acceptance in recent years [9]. MI-E devices simulate a natural cough by using positive airway pressure to insufflate the lung (via a face mask), followed by a rapid shift to negative pressure and lung exsufflation.

Techniques to promote cough and enhance mucociliary clearance have been mainstay treatments of NMD patients during episodes of acute respiratory deterioration and are now being used proactively [6-8,10,11]. Multiple studies have demonstrated the clinical benefit of MI-E in patients living with NMD [6-8,10,12]. In adults with NMD, MI-E results in significantly increased peak cough flow [13-17], decreased treatment time [18], improved oxygen saturation and decreased dyspnoea [16], and the potential to decrease mechanical ventilation time [19]. In children, MI-E has been shown to significantly increase peak cough flow [18] and is well tolerated and safe [20,21]. Further studies comparing the use of MI-E in combination with other therapeutic interventions describe the need to monitor respiratory status when making respiratory management decisions [22].

Limited studies have examined the impact of home use of MI-E devices on health service utilisation. Moran et al., conducted a retrospective chart review of seven children with NMD using a MI-E device at home and found a significant reduction in inpatient length of stay at six and twelve months following commencement of home use of a MI-E device. The qualitative parental survey highlighted positive benefits of using a MI-E device, in particular the ability to treat many pulmonary exacerbations at home [9]. Guidelines for respiratory management from the British Thoracic Society, developed from a body of evidence including case studies, recommends homecare treatment includes the use of manually-assisted cough techniques such as the MI-E device and is associated with decreased hospital admission for respiratory infection [23].

The current study included both children and adults with a sample size of 37, and had follow up periods up to five years to evaluate the effects of in-home use of a MI-E device, on ED presentations, pulmonary related hospital separations and inpatient length of stay; and assess the self-reported health and lifestyle benefits experienced as a result of home use of a MI-E device in Western Australia.

## Methods

### Patient survey

As part of an outreach and support service program, Muscular Dystrophy Western Australia (MDWA) provided 45 NMD patients access to a MI-E device (CoughAssist™) at home between December 2007 and November 2011. An allied health professional provided instruction to patients and/or their families and carers on using the MI-E device. Patients then used the MI-E device at home throughout the study period and were able to seek assistance for using the device from MDWA or other health services when required.

Each study participant, or their parent/guardian for participants under the age of 18 years, completed a self-administered paper based questionnaire. The survey instrument allowed for the collection of qualitative data regarding the use of the MI-E device, the self-assessed effects on participant’s respiratory function and satisfaction with the device. The survey instrument can be found in Additional file 1.

### Health record linkage

An ad-hoc linkage between participants and three DOHWA administrative datasets was performed by the Data Linkage Unit, DOHWA using the probabilistic linkage method [24]. Participants’ hospital inpatient and emergency department records were extracted from the Hospital Morbidity Data System (HMDS) between 1988–2012 and the Emergency Department Data Collection (EDDC) between 2005–2012. The death dataset of the Registry of Birth, Deaths and Marriages was used to determine whether any participants died during the study period.

An intervention study design was used to compare health service use pre and post commencing home use of the MI-E device. Respiratory related International Classification of Diseases (ICD), Tenth Revision, Australian Modification (ICD-10-AM) and ICD Ninth Revision, Clinical Modifications (ICD-9-CM) codes were used for this analysis based on advice from a respiratory specialist and a medical coding specialist. The codes are listed in Additional file 1.

### Statistical analysis

All three outcome measures (dependent variables) were modelled using a negative binomial regression (to allow for overdispersion) with a log link function fitted in SAS version 9.3 (SAS Institute, Cary, NC, USA). LOS was calculated as time from admission to separation minus leave days. The explanatory variables in the model were time period, sex and access to a MI-E device at home (the intervention). Time period was used as a surrogate variable for disease progression.

For the HMDS data extracted for the 25 years between 1988–2012, the variable for time period was constructed in the form of 25 one year intervals relative to the intervention date (the date at which an individual received their MI-E device) such that there were up to a total of 20 intervals before intervention and up to five intervals after intervention. For ED data, extracted for the eight years between 2005–2012, the variable for time period was constructed in the form of eight one year intervals relative to the intervention date, totalling up to three intervals before intervention and up to five intervals after intervention. Our analysis took into account the amount of time clinical data was available for each person. For every one year period, the total number of person days contributed for the year by each person was calculated. As participants began using the MI-E device at different times, person days contributed were not equal for all periods for all cohort members. Logarithmic person days were modelled as an offset term that is equivalent to modelling the dependent variable as a rate model [25].

This study was approved by the Human Research Ethics Committee of the DOHWA.

## Results

### Patient survey

Thirty seven of the 45 patients who were invited to participate in this study consented to take part in the study; a participation rate of 82%. Table 1 shows the demographic characteristics and results from the survey. The majority of participants (73%) were male, the mean age of participants at time of receiving the machine was 19.8 years (range 1–59 years). The participants used the MI-E devices for 0.1 - 4.0 years, with a mean duration of 2.3 years (median 2.5 years). More than 80% of participants resided in the metropolitan area. Of 37 cases, more than half (55.6%) had a diagnosis of muscular dystrophy (MD), of which included approximately three quarters of the male participants. The remaining participant’s (male and female) had a variety of NMD diagnoses including spinal muscular atrophy (SMA), limb girdle MD, congential myopathies, arthrogryposis multiplex congenital and congenital MD.

**Table 1:** **Demographic characteristics of study participants and survey responses**

		**Respondents**	
		**Number**	**(%)**
**Mean age in years**		19.8	
**(range)**		(1–59)	
**Gender**	Male	27	(73.0)
	Female	10	(27.0)
**Residency**	Metropolitan	32	(86.5)
	Rural	5	(13.5)
**Frequency of home MI-E device use**	Daily	17	(46.0)
	Weekly	10	(27.0)
	Fortnightly	1	(2.7)
	Monthly	3	(8.1)
	Rarely	6	(16.2)
**Administration of MI-E device**	Self-administered	3	(8.1)
	With support from family member and/or support worker	34	(91.9)
**MI-E device has ever been used to resolve a choking episode**	Yes	12	(32.4)
	No	25	(67.6)

The majority of participants (92%) reported that the MI-E device was being administered with support either from a family member or a support worker. The majority of participants used the device either daily (46%) or weekly (27%). One third reported they had used the MI-E device to resolve a choking episode. Thirty three (88%) participants either strongly agreed or agreed that home MI-E had improved their/their child’s overall respiratory health. Over 91% either strongly agreed or agreed that they were satisfied with the home use MI-E device and over 94% reported that they would recommend the use of a home MI-E device to other people living with NMD.

Analysis of qualitative responses indicates that participants perceived that use of a home MI-E device had led to improvements of their/their child’s respiratory function, prevented respiratory infections and complications and allowed the pro-active management of respiratory events. The device’s ease of use, the ability to manage health care needs at home and the reduction in hospitalisations were also reported as positive features. Finally, participants reported that having access to a MI-E device at home provided peace of mind that episodes of choking could be managed quickly and effectively, with some participants describing the device as a “lifesaver”.

Only one-third of participants reported negative features of using the MI-E device. The most commonly reported negative features were the size and weight of the machine and the time required to administer the device.

### Health record linkage

Links to hospital morbidity data were found for all 37 study participants, however, only 23 (62%) study participants were admitted to hospital for a respiratory related condition over the study period. Among those participants who had been admitted to hospital for a respiratory related condition, the mean and median total number of respiratory hospital separations between 1988–2012 was 3.1 and 2.0 respectively.

Links to ED data were found for 29 (78%) study participants. The mean and median number of total ED presentations amongst these study participants between 2005–2012 were 7.1 and 6.0 presentations respectively. No links to death data were found for any participant, indicating that none of the participants in the study cohort died during the study period.

**Figure 1:**
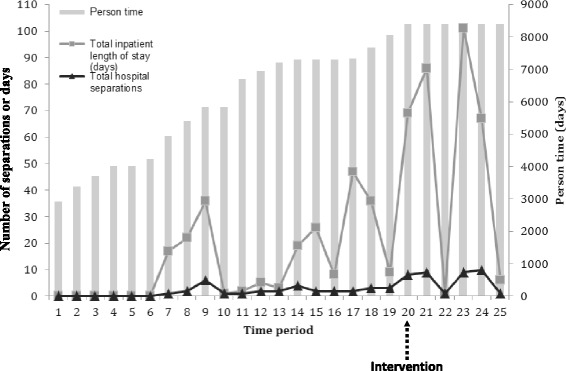
Hospital separations, inpatient LOS and person time contributed by time period.

**Figure 2:**
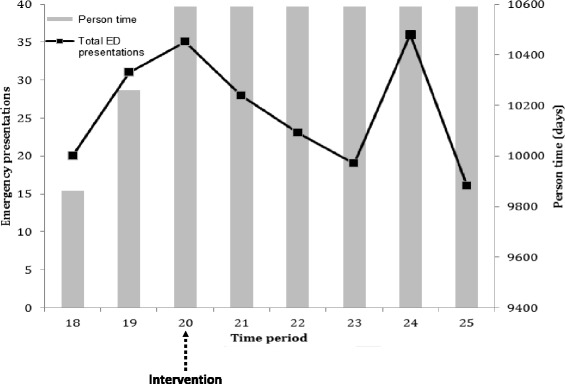
Emergency department presentations and person time contributed by time period.

Figure 1 illustrates the hospital separations, inpatient LOS and person time contributed by time period for the study cohort. The person time increases over the study period as additional participants enter the cohort and remains constant after intervention (time period 20). While there is an overall increasing trend, although not statistically significant, in both hospital separations and inpatient LOS over the study period, there is considerable fluctuation from one time period to the next. The person time for ED presentation also increased until intervention (Figure 2). There was an overall decreasing trend, although not statistically significant, in ED presentations after intervention.

The relative risk (RR) of hospital separations, inpatient LOS and ED presentation before and after intervention were shown in Table 2. Males with NMD had lower risk compared to females with NMD in all 3 outcomes. After adjusting for gender, person years and time period, the risk of hospitalisations (RR = 1.82, 95% CI = 0.76-4.38) and LOS (RR = 2.83, 95% CI = 0.52-15.49) at pre-intervention were higher than post-intervention, but this was not statistically significant. The home use of an MI-E device was shown to be significantly associated with reduced ED presentations (RR = 1.76, 95% CI =1.10-2.84).

**Table 2:** **Relative risk of hospital separations, inpatient LOS and ED presentations**

		**Hospital separations**	**Inpatient LOS**	**ED presentations**
		**RR (95% CI)**	**RR (95% CI)**	**RR (95% CI)**
**Gender**	Male	0.44 (0.24 - 0.8)	0.21 (0.07 - 0.63)	0.42 (0.31 - 0.58)
	Female	1.0	1.0	1.0
**Intervention**	Pre	1.82 (0.76 - 4.38)	2.83 (0.52 - 15.49)	1.76 (1.10 - 2.84)
	Post	1.0	1.0	1.0
**Time period**		1.10 (1.04 -1.17)	1.15 (1.01 – 1.27)	1.05 (0.95 – 1.16)

## Discussion

This study demonstrated that using MI-E devices at home improves self-reported health and lifestyle factors including maintaining respiratory function and preventing respiratory infections; the ability to manage respiratory illness at home. Participants reported they would recommend the program to other patients with NMD. These survey findings are congruent with those reported by Moran et al. [9].

Patients and their families/carers reported the device provided reassurance and peace of mind that acute choking episodes could be managed quickly and effectively. This is particularly important considering choking is a significant cause of death in NMD patients [14].

Patients had almost two and three times higher hospitalisations and LOS at pre-intervention, respectively, but the results are not statistically significant. The non-significant results may be explained by the fact that 27% of participants did not use the device regularly (less than weekly) and the limited time frame in which participants were followed after receiving a MI-E device. The regularity of home MI-E use among NMD patients may depend on their changing health needs and disease progression. Advantages of frequent use include not only the maintenance of health but familiarity with the device, resulting in patients and their families/carers feeling more confident in using the device during an acute respiratory episode, such as choking [21,9]. Hence, regular access to this equipment by the provision of continual home access programs may be more effective than programs that provide only intermittent access to MI-E devices.

The use of a MI-E device was significantly associated with a reduction of NMD patients’ ED presentations. It is not known whether all of the ED presentations both pre and post intervention were respiratory related since the ED data had missing values in the diagnosis field. Nevertheless, the reduction in ED presentations is a desirable outcome for patients and the health system to reduce patient's and family’s stress and overcrowding in emergency departments [26,27].

The increasing trend over time in hospital separations and inpatient LOS are most likely explained by two factors: 1) the gradual increase in the number of participants in the study cohort over the study period and 2) disease progression among study participants that results in a deterioration of respiratory function and an increase in respiratory morbidity and therefore hospitalisation. Additionally, the variation of participants age and disease status may well have masked any underlying trend.

The limitations of this study are characteristic of the common logistical and statistical challenges of studies evaluating medical interventions for rare diseases mainly attributed to small patient numbers [28]. The lack of control group is a limiting but inevitable factor due to the nature of the disease group and the small population of people living with NMD in WA. This makes finding suitable controls matched on age, sex and diagnosis impracticable. Due to the low subject numbers, it is not possible to stratify subjects further on disease types, regularity of MI-E device use, children and adults. There is a gender imbalance which is also due to the low sample size. The age range of patients in this study has resulted in the variability in disease progression and limited the potential to reach significance. For the analysis, time period was used as a surrogate variable for disease progression, however, this has limitations because of the individual variability in disease progression.

Finally, information about self-reported respiratory health and disease was not available prior to home use of a MI-E device as the patient survey was a retrospective study. This study was therefore limited to questioning participants about their perspectives regarding changes in their overall respiratory health since commencing the use of a MI-E device.

## Conclusion

This is the first report examining the impact of home use of MI-E on hospital service use in a study population of both adults and children with NMD. Study results found that in-home use of a MI-E device was associated with a significant reduction in ED presentations and associated with a number of perceived health and lifestyle benefits. Future research requires matched controls to people with NMD who are not using a MI-E device and longer follow-up periods after commencement of home use of a MI-E device. Participants need better education on the regular use of the machine to maximise the benefit. In addition, the use of validated quality of life tools as suggested by Moran et al. [9] or a more in-depth, exploratory approach to qualitatively examine the impact of home use of a MI-E device would greatly enhance research to assist in planning of health services and home assistance.

Considering the already complex care needs of people living with NMD, it is important that medical technologies introduced into patient homes do not result in an unacceptable increase in burden of care.

## Additional file

Additional file 1:
**Included are data of hospital separations, inpatient LOS and ED presentations, respiratory-related codes and the survey instrument.** Data of hospital separations, inpatient LOS and total time contributed by sex and time period **(Table S1)**, and intervention and time period **(Table S2)**. Data of ED presentations and total time contributed by sex and time period **(Table S3)**, and intervention and time period **(Table S4)**. **Table S5** lists the respiratory related International Classification of Diseases (ICD), Tenth Revision, Australian Modification (ICD-10-AM) and ICD Ninth Revision, Clinical Modifications (ICD-9-CM) codes used for this analysis based on advice from a respiratory specialist and a medical coding specialist. **Table S6** is the survey instrument used for the collection of qualitative data regarding the use of the MI-E device, the self-assessed effects on participant’s respiratory function and satisfaction with the device.

## Abbreviations

DMD: Duchenne muscular dystrophy
DOHWA: Department of Health Western Australia
ED: Emergency Department
EDDC: Emergency Department Data Collection
HMDS: Hospital Morbidity Data System
LOS: Length of stay
MDWA: Muscular Dystrophy Western Australia
MI-E: Mechanical insufflation-exsufflation
NMD: Neuromuscular Disorders
RR: Relative Risk
WA: Western Australia
