# Supplementary material for: Use of mechanical airway clearance devices in the home by people with neuromuscular disorders: effects on health service use and lifestyle benefits
Source: Orphanet J Rare Dis. 2015 May 6;10:54. doi: 10.1186/s13023-015-0267-0 (PMC4432957; doi:10.1186/s13023-015-0267-0)
Supplement: Additional file 1: — Included are data of hospital separations, inpatient LOS and ED presentations, respiratory-related codes and the survey instrument. Data of hospital separations, inpatient LOS and total time contributed by sex and time period (Table S1), and intervention and time period (Table S2). Data of ED presentations and total time contributed by sex and time period (Table S3), and intervention and time period (Table S4). Table S5 lists the respiratory related International Classification of Diseases (ICD), Tenth Revision, Australian Modification (ICD-10-AM) and ICD Ninth Revision, Clinical Modifications (ICD-9-CM) codes used for this analysis based on advice from a respiratory specialist and a medical coding specialist. Table S6 is the survey instrument used for the collection of qualitative data regarding the use of the MI-E device, the self-assessed effects on participant’s respiratory function and satisfaction with the device. [file 13023_2015_267_MOESM1_ESM.docx]

**Table S1**. Hospital separations, inpatient LOS and total time contributed by sex and time period

|  | **Male** | | | **Female** | | |
| --- | --- | --- | --- | --- | --- | --- |
| **Period** | **Separations** | **Length of Stay** | **Person time** | **Separations** | **Length of Stay** | **Person time** |
| 1 | 0 | 0 | 2556.75 | 0 | 0 | 365.25 |
| 2 | 0 | 0 | 3011.75 | 0 | 0 | 365.25 |
| 3 | 0 | 0 | 3348 | 0 | 0 | 365.25 |
| 4 | 0 | 0 | 3652.5 | 0 | 0 | 365.25 |
| 5 | 0 | 0 | 3652.5 | 0 | 0 | 365.25 |
| 6 | 0 | 0 | 3744 | 0 | 0 | 486.75 |
| 7 | 1 | 17 | 4201.5 | 0 | 0 | 730.5 |
| 8 | 1 | 5 | 4659 | 1 | 17 | 730.5 |
| 9 | 2 | 3 | 5113.5 | 4 | 33 | 730.5 |
| 10 | 1 | 1 | 5113.5 | 0 | 0 | 730.5 |
| 11 | 1 | 2 | 5113.5 | 0 | 0 | 1581.75 |
| 12 | 2 | 5 | 5113.5 | 0 | 0 | 1826.25 |
| 13 | 2 | 3 | 5387.75 | 0 | 0 | 1826.25 |
| 14 | 0 | 0 | 5478.75 | 4 | 19 | 1826.25 |
| 15 | 0 | 0 | 5478.75 | 2 | 26 | 1826.25 |
| 16 | 1 | 3 | 5478.75 | 1 | 5 | 1826.25 |
| 17 | 1 | 45 | 5509 | 1 | 2 | 1826.25 |
| 18 | 2 | 35 | 5844 | 1 | 1 | 1826.25 |
| 19 | 3 | 9 | 5844 | 0 | 0 | 2223.75 |
| 20 | 4 | 38 | 5844 | 4 | 31 | 2556.75 |
| 21 | 3 | 53 | 5844 | 6 | 33 | 2556.75 |
| 22 | 1 | 1 | 5844 | 0 | 0 | 2556.75 |
| 23 | 5 | 43 | 5844 | 4 | 58 | 2556.75 |
| 24 | 8 | 53 | 5844 | 2 | 14 | 2556.75 |
| 25 | 1 | 6 | 5844 | 0 | 0 | 2556.75 |

**Table S2**. Hospital separations, inpatient LOS and total time contributed by intervention and time period

|  | **Intervention** | | | **No Intervention** | | |
| --- | --- | --- | --- | --- | --- | --- |
| **Period** | **Separations** | **Length of Stay** | **Person time** | **Separations** | **Length of Stay** | **Person time** |
| 1 | 0 | 0 | 0 | 0 | 0 | 2922 |
| 2 | 0 | 0 | 0 | 0 | 0 | 3377 |
| 3 | 0 | 0 | 0 | 0 | 0 | 3713.25 |
| 4 | 0 | 0 | 0 | 0 | 0 | 4017.75 |
| 5 | 0 | 0 | 0 | 0 | 0 | 4017.75 |
| 6 | 0 | 0 | 0 | 0 | 0 | 4230.75 |
| 7 | 0 | 0 | 0 | 1 | 17 | 4932 |
| 8 | 0 | 0 | 0 | 2 | 22 | 5389.5 |
| 9 | 0 | 0 | 0 | 6 | 36 | 5844 |
| 10 | 0 | 0 | 0 | 1 | 1 | 5844 |
| 11 | 0 | 0 | 0 | 1 | 2 | 6695.25 |
| 12 | 0 | 0 | 0 | 2 | 5 | 6939.75 |
| 13 | 0 | 0 | 0 | 2 | 3 | 7214 |
| 14 | 0 | 0 | 0 | 4 | 19 | 7305 |
| 15 | 0 | 0 | 0 | 2 | 26 | 7305 |
| 16 | 0 | 0 | 0 | 2 | 8 | 7305 |
| 17 | 0 | 0 | 0 | 2 | 47 | 7335.25 |
| 18 | 0 | 0 | 0 | 3 | 36 | 7671.25 |
| 19 | 0 | 0 | 0 | 3 | 9 | 8067.75 |
| 20 | 0 | 0 | 0 | 8 | 69 | 8400.75 |
| 21 | 0 | 0 | 730.5 | 9 | 86 | 7670.25 |
| 22 | 1 | 1 | 3652.5 | 0 | 0 | 4748.25 |
| 23 | 4 | 60 | 4748.25 | 5 | 36 | 3625.5 |
| 24 | 7 | 47 | 6574.5 | 3 | 20 | 2556.75 |
| 25 | 1 | 6 | 8400.75 | 0 | 0 | 0 |

**Table S3**. ED presentations and total time contributed by sex and time period

|  | **Male** | | **Female** | |
| --- | --- | --- | --- | --- |
| **Period** | **ED presentations** | **Person time** | **ED presentations** | **Person time** |
| 18 | 14 | 8035.5 | 6 | 1826.25 |
| 19 | 16 | 8035.5 | 15 | 2225.25 |
| 20 | 15 | 8035.5 | 20 | 2556.75 |
| 21 | 16 | 8035.5 | 12 | 2556.75 |
| 22 | 15 | 8035.5 | 8 | 2556.75 |
| 23 | 10 | 8035.5 | 9 | 2556.75 |
| 24 | 23 | 8035.5 | 13 | 2556.75 |
| 25 | 12 | 8035.5 | 4 | 2556.75 |

**Table S4**. ED presentations and total time contributed by intervention and time period

|  | **Intervention** | | **No Intervention** | |
| --- | --- | --- | --- | --- |
| **Period** | **ED presentations** | **Person time** | **ED presentation** | **Person time** |
| 18 | 0 | 0 | 20 | 9861.75 |
| 19 | 0 | 0 | 31 | 10260.75 |
| 20 | 0 | 0 | 35 | 10592.25 |
| 21 | 3 | 1095.75 | 25 | 9496.5 |
| 22 | 3 | 4748.25 | 20 | 5844 |
| 23 | 11 | 6939.75 | 8 | 3652.5 |
| 24 | 30 | 8766 | 6 | 1826.25 |
| 25 | 16 | 10592.25 | 0 | 0 |

**Table S5**. ICD-9-CM and ICD-10-AM codes

| 463 | Acute tonsillitis |
| --- | --- |
| 465.9 | Acute uri nos |
| 474 | Chronic tonsillitis |
| 474.12 | Hypertrophy adenoids |
| 480.1 | Resp syncyt viral pneum |
| 486 | Pneumonia, organism nos |
| 493.9 | Asthma, unspecified |
| 493.9 | Asthma w/o status asthm |
| 507 | Food/vomit pneumonitis |
| 518 | Pulmonary collapse |
| 769 | Respiratory distress syn |
| 770.6 | Nb transitory tachypnea |
| J03.9 | Acute tonsillitis unspecified |
| J06.9 | Acute URTI unspecified |
| J09. | Influenza dt id avian influenza virus |
| J10.1 | Influenza w oth resp manif oth virus id |
| J12.1 | Respiratory syncytial virus pneumonia |
| J12.8 | Other viral pneumonia |
| J12.9 | Viral pneumonia unspecified |
| J18.0 | Bronchopneumonia unspecified |
| J18.9 | Pneumonia unspecified |
| J21.0 | Ac bronchiolitis dt resp syncytial virus |
| J22. | Unsp acute lower respiratory infection |
| J35.0 | Chronic tonsillitis |
| J40. | Bronchitis not spec as acute or chronic |
| J96.0 | Acute respiratory failure |
| J96.9 | Respiratory failure unspecified |
| J98.8 | Other specified respiratory disorders |
| R05. | Cough |
| R06.8 | Other and unsp breathing abnormalities |
| R09.2 | Respiratory arrest |

**Table S6**. CoughAssist Program Evaluation Questionnaire

1. Age (please state)

2. Gender

Male

Female

3. Are you of Aboriginal or Torres Strait Islander origin?

Yes

No

4. What is your postcode? (please state)

5. What is your medical diagnosis? (please state)

6. When did you receive the CoughAssist machine from Muscular Dystrophy WA?

(if unsure, please leave blank) Date (month/year)

7. Have you contacted Muscular Dystrophy WA for support in using the CoughAssist machine?

Yes Go to Question 7a

No Go to Question 8

7a.How often have you contacted Muscular Dystrophy WA for support in using the CoughAssist machine?

Once

2-3 times

4-5 times

6-7 times

8-9 times

10 or more times

Unsure

8. Have you contacted another organisation for support in using the CoughAssist machine?

Yes Go to Question 8a

No Go to Question 9

8a.Please state the name of the organisation you have contacted for support in using the CoughAssist machine?

9. Who administers the CoughAssist machine to you?

I use the device myself

A family member

A support worker

Other (please state)

10. Why did you receive the CoughAssist machine?

It was recommended (Go to Question 10a)

Other (please state) (Go to Question 11)

10a.Who recommended you should use the CoughAssist machine?

Princess Margaret Hospital

Sir Charles Gairdner Hospital

Royal Perth Hospital

Muscular Dystrophy WA

Rocky Bay

Disability Services Commission

11. How often do you use the CoughAssist machine when you are well?

Daily

Weekly

Fortnightly

Monthly

Rarely

12. If you are unwell (e.g cold, flu) on average, how many times would you use the CoughAssist machine per day? (please state) times

13. Have you ever used the CoughAssist machine to resolve a choking episode?

Yes

No

14. Since using the CoughAssist machine, have you:

Yes

No

a) Had a respiratory condition / cold and managed it yourself without going to the doctor or Emergency Department?

b) Been to the doctor or Emergency Department for a respiratory condition / cold but not required hospitalization?

c) Been admitted to hospital for a respiratory condition / cold?

If yes, go to Question 14a. Otherwise, go to Question 15.

14a. How many times have you been admitted to hospital in Western Australia for a respiratory condition since using the CoughAssist machine?

Once

2-3 times

4-5 times

6-7 times

8-9 times

10 or more times

Unsure

15. Please indicate how much you agree or disagree with the following:

Strongly Agree

Agree

Neither

Disagree

Strongly Disagree

a) The CoughAssist machine has improved my respiratory health.

b) I am satisfied with the CoughAssist machine.

c) I would recommend the CoughAssist machine to others with a neuromuscular condition.

16. What do you believe are the positive features of the CoughAssist Machine?

17. What do you believe are the negative features of the CoughAssist machine?

18. Have you previously used a CoughAssist machine but have since returned it to Muscular Dystrophy WA?

Yes Go to Question 18a

No Go to Question 19

18a.If yes, please state why you returned the CoughAssist machine to Muscular Dystrophy WA

19. Do you have any other comments about the CoughAssist machine or the Outreach and Support Services Program provided by Muscular Dystrophy WA?
